# Retinal Fundus Image Registration via Vascular Structure Graph Matching

**DOI:** 10.1155/2010/906067

**Published:** 2010-09-07

**Authors:** Kexin Deng, Jie Tian, Jian Zheng, Xing Zhang, Xiaoqian Dai, Min Xu

**Affiliations:** ^1^School of Electronic Engineering, Xidian University, Xi'an, Shanxi 710071, China; ^2^Institute of Automation, Chinese Academy of Sciences, Beijing 100190, China

## Abstract

Motivated by the observation that a retinal fundus image may contain some unique geometric structures within its vascular trees which can be utilized for feature matching, in this paper, we proposed a graph-based registration framework called GM-ICP to align pairwise retinal images. First, the retinal vessels are automatically detected and represented as vascular structure graphs. A graph matching is then performed to find global correspondences between vascular bifurcations. Finally, a revised ICP algorithm incorporating with quadratic transformation model is used at fine level to register vessel shape models. In order to eliminate the incorrect matches from global correspondence set obtained via graph matching, we proposed a structure-based sample consensus (STRUCT-SAC) algorithm. The advantages of our approach are threefold: (1) global optimum solution can be achieved with graph matching; (2) our method is invariant to linear geometric transformations; and (3) heavy local feature descriptors are not required. The effectiveness of our method is demonstrated by the experiments with 48 pairs retinal images collected from clinical patients.

## 1. Introduction

In this paper, we proposed a novel graph-based registration framework, namely GM-ICP, to align pairwise retinal images. It consists of three independent steps. First, the retinal vessels are automatically detected and represented as vascular structure graphs; a graph matching (GM) is then performed to find global correspondences between vascular bifurcations; finally, the ICP algorithm is used at fine level to register vessel shape models.

The image registration techniques become increasingly important in clinical human retina diseases diagnosis and treatment. There are large demands for fast and fully automatic algorithms to register two or more retinal images which were taken at different times, from different views, and by different sensors. Accurate registration of intra- and intermodality retinal images is required for information integration. Register a sequence of images of the same retina can give a complete view of the retinal fundus region which may lead to early diagnosis and treatment of AIDS-CMV retinopathy [1]. In laser retinal surgery, the treatment of exudative AMD may cause highly 50% recurrence rate of CNV (Choroidal Neovascularization) due to inaccurate localization of the laser in fovea area. Real-time image registration method can be used to assist ophthalmologists with avoiding possible damages during the surgery [2].

Retinal images registration is a challenging task [3]. First, the retina is a curved surface; nonlinear deformation may occur when using a weak-perspective uncalibrated camera. Second, image overlap may be small due to large viewpoint change between images. Third, retinal images may have large textureless regions with uneven illumination which make the extraction of retinal vessels very difficult. Fourth, two images taken long time apart or from diseased eyes can have physical changes in both structure and color of the retina.

In the past ten years, many techniques have been proposed to solve the retinal image registration problem in different ways. Generally, these methods can be classified into two categories: vessel-based and nonvessel-based methods.

The nonvessel-based methods refer to a class of techniques that do not make use of retinal vessels directly during image registration. These methods can be further subdivide into two classes: intensity-based and image descriptor-based methods. The intensity-based techniques usually rely on image intensities and gradients, the registration is carried out through optimizing a certain similarity function, such as mean-square error, mutual information [4], and cross-correlation of images. Various of optimization method can be used for finding global optimum of the cost function, including downhill simplex, simulated annealing [4, 5], genetic algorithms [5], and so forth. In [6], a multiscale elastic registration scheme was proposed to take into account retinal intensity variants based on optical flows. The main limitations of intensity-based methods lie in two aspects: (1) these methods are highly reliant on the consistent intensities in two images and tend to fail due to the presence of nonuniform illumination and large textureless regions; (2) the optimization may have huge searching space so that the computational cost becomes a bottleneck for intensity-based methods to be applied in clinic.

The registration methods with image descriptors [7–9] are feature-based and become popular recently. Instead of optimizing a similarity function with whole image intensities, these methods extract local invariant descriptors as image features. And then, image registration is equivalent to finding correspondences between two feature sets. In [8], a generalized dual-bootstrap iterative closest point (GDB-ICP) was proposed, in which SIFT local descriptor is used and the alignment process is driven by two types of keypoints: corner points and face points. In [7], to deal with multimodal registration problem, an edge-driven DB-ICP algorithm was developed by enriching the keypoint descriptor with shape context using edge points. In [9], a new salient feature region descriptor was proposed for low quality retinal images registration.

The vessel-based methods utilize retinal vascular features as a basis for image matching. Currently, most of the retinal image registration approaches are vessel-based [3, 10–15], because the retinal vessels are believed to be the most appropriate representation for retina. In general, the vessel-based methods consist of two independent steps: vessel segmentation and vascular feature-based registration. Once vessels were extracted, establishing point correspondences between segmented vessels is crucial. In [13], a dual-bootstrap iterative closest point (DB-ICP) algorithm was introduced to match vascular centerlines. It started with some initial low-order estimations and iteratively refined the results by expending the bootstrap regions with higher-order transformation model. In order to make the matching robust to mismatches between feature points, hierarchical transformation models were employed in [3]. In [11], a hybrid retinal image registration approach is proposed by combining both intensity-based and vessel-based methods. To achieve both global and local alignments, an elastic matching scheme is used in [12] based on reconstructed vascular trees. Usually, the vessel-based method are supposed to be more reliable than nonvessel-based method because the vascular features are sufficient (but not too many) and accurate as landmarks and preferable spread over whole image region. The main drawback of vessel-based methods is the local convergence problem when initial misalignment is large and mass of segmentation noises exist.

**Figure 1:**
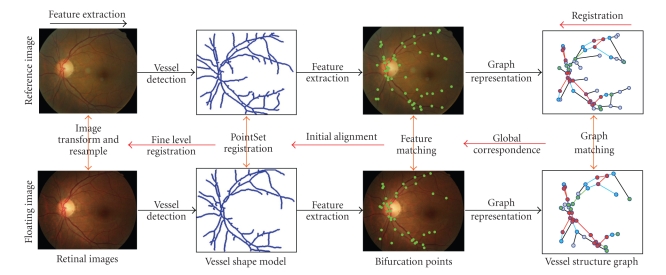
Hierarchical retinal image feature extraction and registration framework proposed in this paper. From left to right is the process of feature extraction, including vessel detection, vascular bifurcations extraction, and graph-based representation. From right to left represents the two-step registration procedure, consisting of graph matching and ICP-based vessel shape model registration.

In this paper, we study retinal image registration following vessel-based approaches. Inspired by the fact that a retinal image may contain some unique geometric structures within its vascular trees. We consider that the correspondence problem is not simply a one-to-one mapping problem between point sets but rather a pairwise (structure) matching problem between two separate graphs. A brief overview of our approach is illustrated in Figure 1, in which the retinal feature extraction is preformed at first to generate a graph representation of the vessel networks. Afterward, a graph matching-based registration is performed to achieve both globally and locally alignment of the retinal images.

To our knowledge, the graph-based approaches are relatively rare in the field of retinal image registration. Related work proposed recently were reported in [16, 17], where a graph transformation matching (GTM) algorithm was developed for retinal image registration and vascular characterization. However, their method is limited in three aspects: (1) a good initial guess of correspondences is crucial to GTM; (2) the number of graph nodes must be equal; and (3) the vessels are detected in a semi-automatic way.

There are three major contributions in this paper. Firstly, we demonstrated that the retinal fundus image registration problem can be efficiently solved within a graph-matching framework. Accordingly, we innovatively represent the retinal vascular features as an undirected graph model and a normalized vessel path distance measure is defined to characterize local similarities between two graph edges. Secondly, we proposed a hybrid registration framework that integrates graph matching and the ICP algorithm together to work with a hierarchy of transformation models. Thirdly, we proposed a structure-based sample consensus (STRUCT-SAC) algorithm to eliminate incorrect correspondences from graph matching results.

The remainder of this paper is organized as follows. The feature extraction methods are described in Section 2. The GM-ICP registration framework is introduced in Section 3. Experiment results are shown in Section 4. Finally, we outline our conclusions in Section 5.

## 2. Feature Extraction

**Figure 2:**
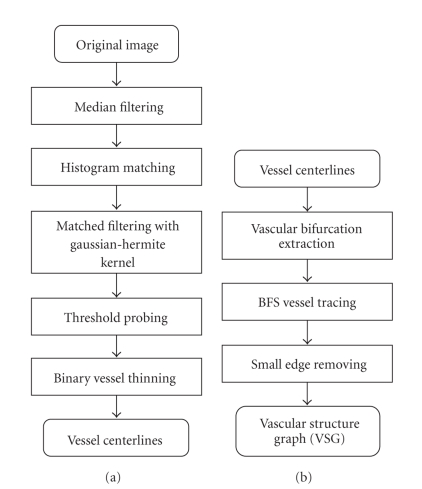
Flowchart of retinal feature extraction method described in this paper. (a) The process of vessel centerline detection. (b) The process of VSG construction.

The key step of GM-ICP is the extraction of invariant image features. This section presents the feature extraction methods used in our registration framework, including vessel centerline detection and graph-based representation. A detailed flowchart of above two algorithms is illustrated in Figure 2.

### 2.1. Vessel Centerline Detection

Retinal vessels are the most unique and prominent anatomical structure in the retina and provide rich information for the localization of distinct features. A variety of techniques has been introduced for retinal vessel extraction, such as the Hessian method [14], the Gabor filter [18], matched filter [19], and parallel edge method [20]. In our implementation, a single-scale matched filter is employed to detect retinal vessels because it provides better responses to thin and low-contrast vessels [19]. Although some multiscale techniques [18] can be used to achieve better vessel segmentation results. However, those methods are quite time consuming and not necessary for the image registration task.

Blood vessels in retinal images usually have poor local contrast so that the matched filter is designed to detect and enhance the local piecewise vessel segments through image convolution. To approximate the vessel profile, Gaussian function is commonly adopted as the kernel of matched filter [19]. However, in some cases, the intensity profile of some wide vessel segments is not Gaussian due to vessel central light reflection effect. Hence, we use a Gaussian-Hermite mixture model described in [21] to approximate local vessel segment structures. The 1-D second-order Gaussian-Hermite kernel is defined as

(1) $$\begin{array}{c} H\left(x\right)=\left(1+a\left(x^{2}-1\right)\right)\frac{1}{2\pi \sigma^{2}}e^{(-x^{2}/2\sigma^{2})}, \end{array}$$

where *a* is a weight parameter, that when *a* = 0, the kernel is Gaussian. In order to detect a vessel oriented in any directions, a set rotated kernels is applied to a fundus image. Because of symmetry, only 0° ~ 180° possible directions are required for computation. At each orientation, the two-dimensional kernel can be represented as two one-dimensional kernels applied in succession according to Cartesian separability rule. Let *I*(*u*, *v*) be the image intensity at position (*u*, *v*), a matched-filter response (MFR) of the input image at orientation *θ* is defined as

(2) $$\begin{array}{c} \text{MFR}\left(u,v,\theta \right)=-\iint_{-\infty }^{\infty }H_{vv}\left(v-x\right)H\left(u-y\right)I\left(x,y,\theta \right)dx\;dy, \end{array}$$

where *H*(*x*) is Gaussian-Hermite kernel in normal direction of *θ*, *H*_*vv*_(*x*) is second-order derivative of the Gaussian-Hermite kernel along the direction of *θ*. To make the implementation easier, the image coordinate is rotated at each orientation instead of rotating convolution kernels. Finally, the matched filer is implemented by maximizing the responses over all orientations at each pixel, which can be expressed as

(3) $$\begin{array}{c} \begin{array}{cc} M\left(u,v\right)=\underset{\theta }{\max\;}\left\{\text{MFR}\left(u,v,\theta \right)\right\}, & \;0^{\circ }⩽\theta <180^{\circ }. \end{array} \end{array}$$

In our implementation, 12 matched filter orientations are used with a 15-degree increment. In order to avoid the influence of image noise, a histogram-match image filtering and a median image filtering was applied before MF vessel detection. After the main vascular structures were detected, we used a threshold probing technique proposed in [22] to distinguish between enhanced vessels and the image background. Finally, vessel thinning is performed to the binary vessel image so that the resulting patterns consist of lines and curves with one pixel wide only.

### 2.2. Graph-Based Representation

Once the retinal vessel centerline (vessel shape) model is calculated, the transformation into a graph is straightforward. We construct a undirected vascular structure graph (VSG) *G*(*V*, *E*, *A*), where each graph node *v*_*i*_ ∈ *V*(1 ⩽ *i*⩽|*V*|) represents a vascular bifurcation point or a vessel segment end point; each graph edge *e*_*i*_ ∈ *E*(1 ⩽ *i*⩽|*E*|) corresponds to a vascular segment between two vascular feature points. The attributes set *A* carries some scalar numbers or vectors to characterize the local vessel segments. In our implementation, two distance measures were assigned to each graph edge: vessel path distance *D*_VP_ and Euclidean distance *D*_*E*_ between two linked graph nodes. The vessel path distance measures the vessel path length between two vascular feature points in a binary vessel centerline image. To make the proposed method invariant to scale, we normalize *D*_VP_ in the following way:

(4) $$\begin{array}{c} A\left(i\right)=\frac{D_{\text{VP}}\left(i\right)}{\left(1/\left|E\right|\right)\sum_{k=1}^{\left|E\right|}D_{\text{VP}}\left(k\right)}, \end{array}$$

likewise for *D*_*E*_. In VSG, all the graph nodes are treated uniformly, we do not distinguish between the type of vertices on anatomical aspects.

The vascular structure graph model is built by following two steps. First, the bifurcations of vessels are extracted using a template matching technique described in [10]. Then, we performed a parallel breadth first search (BFS) on the mono-pixel binary retinal vessel network to build edges among those bifurcation points and vessel segment end points. The vessel path distance is calculated at the same time.

## 3. The GM-ICP Algorithm

In this section, we will introduce the proposed GM-ICP registration framework in details. As mentioned above, we solve retinal image registration problem through a feature-based approach. Hierarchical features were utilized in our method, including retinal vessel shape models, vascular bifurcations and the underlying vascular topological structures, which were extracted previously by using the methods described in section 2. The key problem to register two retinal images in different coordinate positions is to establish correspondences between two feature sets. Then, a closed-form solution [23] can be applied to estimate the transformation parameters directly.

The simplest approach to the one-to-one correspondence problem is to define some similarity measure between two feature points, for example, the Euclidean distance between SIFT descriptors. And then the matching is carried out in a high-dimensional feature space following the nearest neighbor rule. This approach will fail when the ambiguities exist, such as nondiscriminative local appearance or repeated patterns. However, these situations are quite common to retinal images. Another way to match two sets of points is to employ some heuristic techniques, such as ICP [23] or EM [24] algorithm, to iteratively align two feature sets until it converges. These methods could be robust if the initial misalignment is small and the noise ratio is not high. However, the features extracted by using current automatic retinal image segmentation techniques will always contain a mass of noises and outliers. The transformation between two images could also be relatively large. As a result, these methods will often converge to local incorrect positions.

Our method was motivated by the observation that a retinal image may contain some unique geometric structures within its vascular trees. Indeed, we are trying to make use of those distinctive structures and to enforce some geometric consistency between pairs of feature correspondences. Furthermore, we find that the graph-matching approach is a natural and powerful way for doing this task. Thus, we want to obtain points correspondence by taking advantage of structure information and the edge-to-edge comparison mechanism with graph matching. More local invariant and expressive features with relatively low computational costs can also benefit from this.

**Figure 3:**
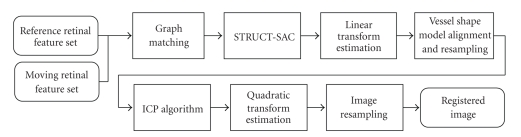
Flowchart of proposed GM-ICP registration algorithm.

The flowchart of the proposed registration algorithm is given in Figure 3. In our implementation, the graduated assignment algorithm is employed to match vascular structure graphs extracted from two retinal images. In order to eliminate wrong matches from global correspondences set obtained via graph matching, a structure-based sample consensus (STRUCT-SAC) algorithm is proposed. This algorithm is more efficient and reliable than RANSAC [25] by replacing the random sampling procedure with a sequential local structure sampling. Once the global transform is known, we use a kdtree-based ICP algorithm to register two vessel shape models to achieve better registration precision.

We use similarity transformation model for global correspondence calculation under the assumption which the apparent motion of the retina is generally rigid [3]. To correct some nonlinear motions in the boundary area with low overlapping images, a quadratic polynomial model is adopted for vessel shape model registration.

### 3.1. Problem Formulations

**Figure 4:**
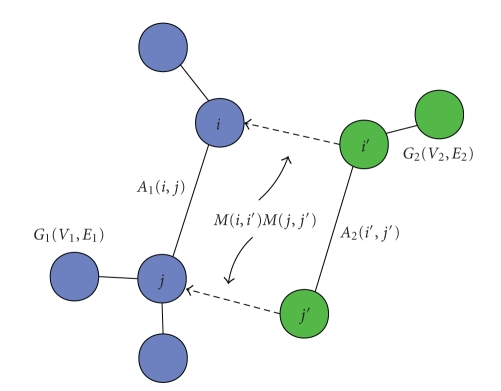
Rectangle rule for subgraph matching, where an assignment is found between the node *i* in *G_1_* and node *i*′ in *G_2_*, likewise for *j* and *j*′.

We consider two vessel structure graphs *G*_1_(*V*_1_, *E*_1_, *A*_1_) and *G*_2_(*V*_2_, *E*_2_, *A*_2_), which were extracted from reference image *I*_1_ and floating image *I*_2_, respectively. Let *N*_1_ = |*V*_1_|, *N*_2_ = |*V*_2_|. We do not assume that *N*_1_ = *N*_2_ because there may be different numbers of features to be matched. The graph-matching problem is to find an optimal assignment between *V*_1_ and *V*_2_ that maximizes the sum of pairwise affinities between edges *e*_1_(*i*, *j*) ∈ *E*_1_ and *e*_2_(*i*′, *j*′) ∈ *E*_2_. This is equivalent to seek a *N*_1_ × *N*_2_ assignment matrix *M* to maximize the objective function

(5) $$\begin{array}{c} E=\underset{M}{\max\;}\overset{N_{1}}{\underset{i}{\sum }}\overset{N_{2}}{\underset{i\prime }{\sum }}\overset{N_{1}}{\underset{j}{\sum }}\overset{N_{2}}{\underset{j\prime }{\sum }}M_{ii\prime }M_{jj\prime }W_{ii\prime jj\prime }, \end{array}$$

where *M*_*ii*′_ is equal to 1 if node *v*_*i*_ in *G*_1_ corresponds to node *v*_*i*′_ in *G*_2_. *W* is a *N*_1_ *N*_2_ × *N*_1_ *N*_2_ compatibility matrix. *W*_*ii*′*jj*′_ carries potential matching weights corresponding to the pairs of points (*v*_*i*_, *v*_*j*_) of *G*_1_ and (*v*_*i*′_, *v*_*j*′_) of *G*_2_. A rectangle rule may better illustrate this edge-matching mechanism, which is shown in Figure 4.

The problem (5) can also be reformulated as an Integer Quadratic Programming (IQP) problem. Let us consider *M* as a *N*_1_ × *N*_2_ binary vector *X* = {*x* | *x*_*ii*′_ = 1  if  *i* *i*′ ∈ *M*}. Then, (5) may take the form as

(6) $$\begin{array}{c} E=\underset{X}{\max\;}\;X^{T}WX. \end{array}$$

In order to achieve one-to-one correspondences, an affine constraint is enforced to assignment matrix *M* as well as to assignment vector *X*

(7) $$\begin{array}{c} \sum_{i}M_{ii\prime }=1,\;\;\sum_{i\prime }M_{ii^{\prime }}=1,\;\;M_{ii^{\prime }}\in \left\{0,1\right\}. \end{array}$$

### 3.2. VSG Matching via Balanced Graduated Assignment

Due to its combinatorial nature, to solve the graph matching problem in an exact way could be NP-Hard and it is not applicable to our VSG matching problem. Therefore, we are seeking to find some approximate solutions. There are many approaches to inexact graph matching in the literature, such as the graduated assignment (GA) algorithm [26], spectral relaxation (SM) method [27], graph edit distance-based method [28], and shock graph grammar-based method [29]. Among these methods, the continuous relaxations approaches were thought to be the most successful ones [27].

In this paper, we employed a graduated assignment algorithm originally proposed by Gold and Rangarajan in [26] as our graph-matching framework. Our empirical evaluation shows that the method is efficient and reliable with VSG matching problem and even when outliers are present. The GA method relaxes the discrete IQP into a continuous nonconvex quadratic program (QP). It optimizes a quadratic cost function through minimizing its low-order Taylor expansion around previous solution. The deterministic annealing scheme was employed to find globally optimum solutions. One-to-one correspondence for graph nodes can be guaranteed via Sinkhorn two-way constraint procedure. Thus, (5) can be reformulated as in following expressions under the GA framework

(8) $$\begin{array}{cc} E\left(M\right) & =\underset{M}{\min\;}-\frac{1}{2}\overset{N_{1}}{\underset{i}{\sum }}\overset{N_{2}}{\underset{i\prime }{\sum }}\overset{N_{1}}{\underset{j}{\sum }}\overset{N_{2}}{\underset{j\prime }{\sum }}M_{ii\prime }M_{jj\prime }W_{ii\prime jj\prime }\\ & \;+\frac{1}{\beta }\overset{N_{1}}{\underset{i}{\sum }}\overset{N_{2}}{\underset{i\prime }{\sum }}M_{ii\prime }\left(\log\;\left(M_{ii\prime }+\alpha \right)-1\right)\\ & \;\text{s}.\text{t}.\;\;\forall i^{\prime }\overset{N_{1}}{\underset{i=1}{\sum }}M_{ii^{\prime }}⩽1,\;\forall i\overset{N_{2}}{\underset{i^{\prime }=1}{\sum }}M_{ii^{\prime }}⩽1,\;M_{ii\prime }⩾0, \end{array}$$

where *M* is a doubly stochastic matrix, in which the maximum element in each row and column represents a correspondence between two graphs nodes. The *x*log *x* term in (8) is a smoothing function which can push the minimum of the objective away from the local discrete points. The weighting parameter *α* is introduced to avoid taking the logarithm of zero. The parameter *β* controls the speed of annealing. We define the measure of compatibility *W*_*ii*′*jj*′_ between the links of two graphs as follows:

(9) $$\begin{array}{c} W_{ii\prime jj\prime }=\{\begin{array}{cc} 0,\;E_{1}\left(i,j\right)\;\text{or}\;E_{2}\left(i^{\prime },j^{\prime }\right) & \text{is}\;\text{null},\\ \overset{2}{\underset{r=1}{\sum }}\omega^{r}\left(1-\frac{1}{L^{r}}\left|A_{1}^{r}\left(i,j\right)-A_{2}^{r}\left(i^{\prime },j^{\prime }\right)\right|\right) & \text{otherwise}, \end{array} \end{array}$$

where *L* is the maximum edge weights in both graphs. Two edge weights are involved for pairwise affinity calculation, where *A* ^1^ = *D*_VP_ and *A* ^2^ = *D*_*E*_ as defined previously. *ω* is a weight parameter to balance the influence of two edge distance measures. Empirically, we set *ω* ^1^ = *ω* ^2^ = 0.5

(10) $$\begin{array}{c} L^{r}=\max\; \end{array}\left\{\max\;\left(A_{1}^{r}\left(i,j\right)\right),\max\;\;\left(A_{2}^{r}\left(i^{\prime },j^{\prime }\right)\right)\right\}.$$

From our experiments, we found that the ambiguities in the compatibility matrix may bring down the quality of graph matching in finding global correspondences. For example, one edge may have lots of potential matches in another graph. Such an edge is not discriminative. On the other hand, an edge with small group of potential matches may help to clarify the optimal matching. To this end, we employ a bistochastic normalization technique described in [27] to balance the compatibility matrix (To enhance correct correspondences and to suppress spurious correspondences). The normalization is carried out in three steps. First, the compatibility matrix *W* is represented as a *M*_1_ × *M*_2_ edge similarity matrix *S*, where *M*_1_ = |*E*_1_| and *M*_2_ = |*E*_2_|. Then, the edge similarity matrix *S* is normalized iteratively by using following expressions until convergence

(11) $$\begin{array}{c} \begin{array}{c} S_{ij,i\prime j\prime }^{t+1}=\frac{S_{ij,i\prime j\prime }^{t}}{\sum_{k\prime l\prime }S_{ij,k\prime l\prime }^{t}},\\ S_{ij,i\prime j\prime }^{t+2}=\frac{S_{ij,i\prime j\prime }^{t+1}}{\sum_{kl}S_{kl,i\prime j\prime }^{t+1}}. \end{array} \end{array}$$

After the normalization was finished, the edge similarity matrix *S* is converted back to the compatibility matrix *W*

### 3.3. STRUCT-SAC

The global correspondences obtained by graph matching may contain a certain number of incorrect matches. In some extreme cases, the ratio of wrong matches can be greater than 80%. To eliminate the wrong matches from global correspondences set is crucial to our method. 

The RANSAC algorithm [25] is a well-known method to deal with outliers and has become a standard in the field of computer vision. It first fits model parameters with randomly selected subsets of input data and then evaluates the quality of the parameters on the whole input data. The process is performed repeatedly and terminated when the probability of finding a better model becomes lower than a user-specified threshold. The main drawback of RANSAC is that most of the verified model parameters are incorrect under arbitrary random sampling. This is inefficient and time consuming when dealing with large data sets. 

In this paper, we proposed a local structure-based sample consensus (STRUCT-SAC) algorithm, based on two simple observations:

- correct matches between graph nodes are not isolated;
- most of the correctly matched graph edges are found next to each other.

**Figure 5:**
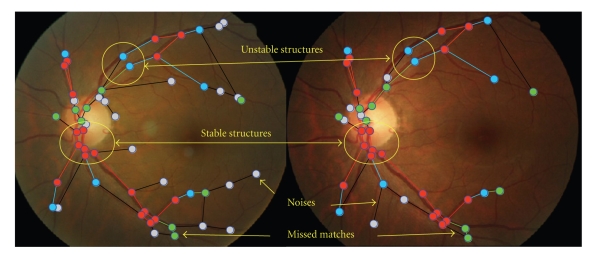
Illustration of the stable and unstable structures in graph matching results, where red color represents stable structures, blue color denotes unstable structures, green color represents missed structures, gray nodes, and black edges are noises.

The phenomenon described above can be interpreted by the property of graduated assignment that it trends to approximately find the maximum common subgraph (MCS) during graph matching. As a result, a good matched edge may have some good matched neighbors to support it. On the other hand, a noise or mismatched edge may influence its neighbors to be mismatched. Furthermore, we thought that the type of correctly matched structures in graph-matching results can be divided into two categories: stable structures and unstable structures (as illustrated in Figure 5). A graph node is stable if it has at least two correctly matched neighbors. An edge between two stable nodes is a stable edge. Otherwise, the structures are unstable. Thus, the goal of our algorithm is simplified to find some stable graph structures. 

**Figure 6:**
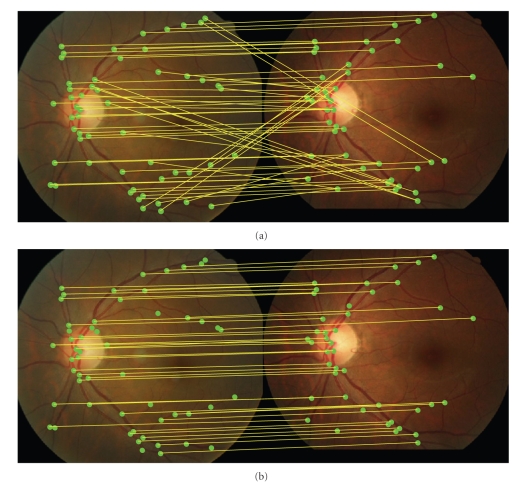
Correspondences between two retinal feature sets after graph matching. (a) Without STRUCT-SAC. (b) With STRUCT-SAC.

The STRUCT-SAC algorithm was motivated by the hypothesize-and-verify idea from RANSAC. However, instead of random sampling, we select subsets of input data in a deterministic way by utilizing the local structures of a sample point. A brief description of STRUCT-SAC is as follows. Repeatedly, select a graph node and with its neighbors as the source subset. Find their corresponding (the correspondence was obtained via graph matching) nodes in other graph as the object subset. (As an option, the RANSAC strategy can be used here to further distinguish the subsets from noisy structures when the number of neighbors is large.) Compute the transformation parameters determined by the selected couples and verify it first with a local geometric consistency test using partial data and then with global model consistency test using whole input data points. The process is terminated when all the potential graph nodes were traversed and the best model parameter with lowest quality score is returned. In our implementation, the search is performed symmetrically and only on the nodes with 2 or 3 degrees. Since these nodes are stable in VSG and provide sufficient information for the estimation of model parameters. The effect of STRAC-SAC is demonstrated in Figure 6.

In local geometric consistency test, we utilize pairwise edge similarity and triplewise orientation similarity of graph nodes to distinguish local structure samples. In global model consistency test, we use sum of squared distance *ɛ*(*θ*) to measure the quality of model parameter *θ* between two point sets, as below:

(12) $$\begin{array}{c} ɛ\left(\theta \right)=\underset{\left(p,q\right)\in C_{\theta }}{\sum }\rho^{2}\left(V_{1}\left(p\right)-T\left(V_{2}\left(q\right),\theta \right)\right), \end{array}$$

where *T* denotes the similarity transformation function, *C*_*θ*_ is a temporary correspondence set determined by transformation parameter *θ*. *ρ* is a Huber kernel [30] to limit the influence of outliers

(13) $$\begin{array}{c} \rho \left(t\right)=\{\begin{array}{cc} \frac{1}{2}t^{2}, & \text{if}\;||t||\leq \sigma ,\\ \sigma ||t||-\frac{1}{2}\sigma^{2}, & \text{otherwise}. \end{array} \end{array}$$

### 3.4. Vessel Shape Model Registration Based on Higher-Order Transformation Model

**Figure 7:**
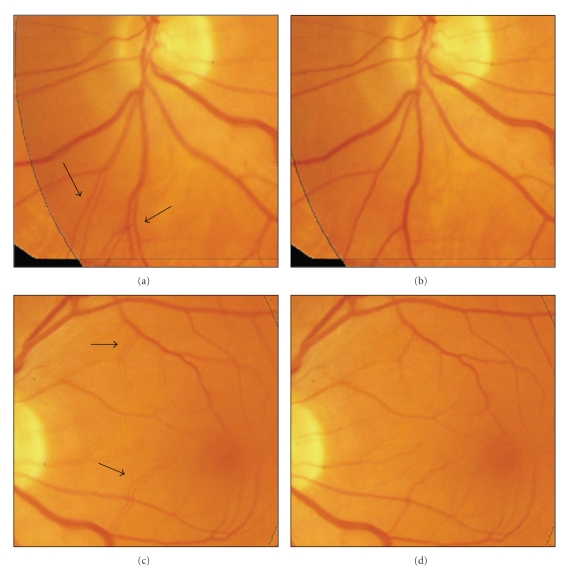
Fused retinal images. (a) and (b) with linear registration, some misalignment vessels were indicated with arrows; (c) and (d) with quadratic registration, vessels are well matched.

Since the retina is spherical and the retinal images captured with weak-perspective cameras may have nonrigid distortion in localized regions, higher-order transformation model is needed for accurate registration. This is more important to the registration tasks with low-overlap retinal images. Figure 7(a) shows two fused retinal images under linear registration where some misalignment vessels were indicated with arrows. In Figure 7(b), these vascular segments are well matched after a quadratic registration.

Our registration algorithms employ a hierarchy of transformation models [3], which is also known as the global to local strategy, to account for the nonlinearities described above. One advantage of hierarchical transformation is that it makes the algorithm robust to the mismatches caused by large interframe motions [3]. In our method, the global registration is achieved via graph matching and STRUCT-SAC with similarity transformation model. At fine (local) level, we use a revised ICP algorithm to register vessel shape (centerline) models. A 12-dimensional second-order polynomial transformation model is employed in the local registration, that it maps a source point (*x*, *y*) in floating vessel model onto the target point (*x*′, *y*′) in reference vessel model as follows:

(14) $$\begin{array}{cc} \left[\begin{array}{c} x\prime \\ y\prime \end{array}\right] & =\left[\begin{array}{ccc} a_{11} & a_{12} & a_{13}\\ a_{21} & a_{22} & a_{23} \end{array}\right]\cdot \left[\begin{array}{c} x^{2}\\ y^{2}\\ xy \end{array}\right]+\left[\begin{array}{cc} a_{14} & a_{15}\\ a_{24} & a_{25} \end{array}\right]\cdot \left[\begin{array}{c} x\\ y \end{array}\right]+\left[\begin{array}{c} a_{16}\\ a_{26} \end{array}\right], \end{array}$$

where *a*_*ij*_ are transformation parameters. Once the correspondence is obtained at each iteration of ICP, the quadratic motion can be estimated in closed-form by using a linear regression method described in [24].

In addition, several improvements are made to the standard ICP algorithm in order to achieve better performance. 

(1) To accelerate the closest point indexing, a kd-tree data structure is applied for underlying point sets modeling; (2) The registration is performed in a multiresolution scheme; (3) A point pair weighting strategy [31] is used to reject inconsistent matches.

## 4. Experiments

In this section, we will evaluate the performance of the proposed GM-ICP registration algorithm in two aspects: (1) saliency of the vascular structure graph, and (2) the overall performance of GM-ICP in retinal image registration. A total of 48 pairs of retinal images collected from clinical patients were involved for the algorithm evaluation. In order to make the evaluation consistent with images in different size, we resampled all test images to approximately 700 × 600  pixel^2^ using bicubic image interpolation.

Our retinal image registration program was designed with matlab and some core algorithms (such as graph matching and vascular bifurcations extraction) were implemented in C-MEX. All the results reported in this paper were obtained on a PC with Intel Core 2 Duo 2.33 GHz CPU and 3 G RAM running under Windows 7 Professional.

### 4.1. Saliency of Vascular Structure Graph

**Figure 8:**
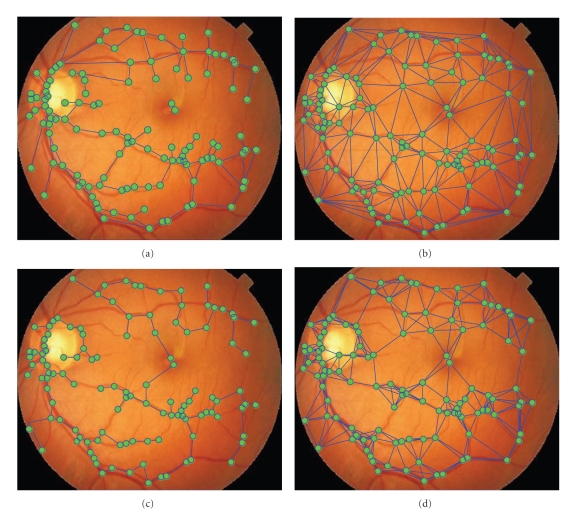
Illustrations for various topological graph structures investigated in our experiment. (a) Vascular structure graph, (b) Delaunay triangulation graph. (c) Minimum spanning tree of DT graph. (d) k-Nearest neighbor graph.

The aim of our first experiment is to show the saliency and performance benefits of vascular structure graphs presented in this paper. Based on the vascular bifurcation points extracted with our methods, several spatial topological graph structures that commonly used in computer vision and graph-matching research were investigated in this study, including Delaunay triangulation graph (DT), minimum spanning tree of DT graph (DT-MST), k-nearest neighbor graph (KNN) [17], and minimum spanning tree of KNN graph (KNN-MST). Some examples for these graph structures are illustrated in Figure 8. The major difference between VSG and other graph representations is the anatomical properties of retinal vessels that VSG characterizes while the other graph representations do not provide.

**Figure 9:**
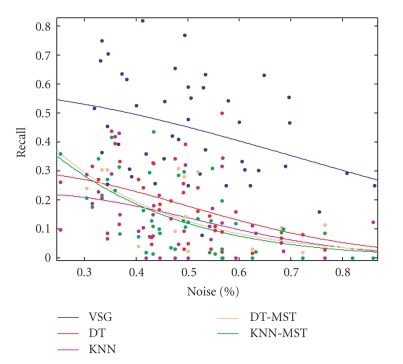
The performance of several topological structures in graph matching under various outlier percentages.

The graph matching results with five different graph structures are shown in Table 1. Three evaluation criterions are presented in our results: the average number of correctly matched graph nodes, the recall rate and the success rate of registration. The recall value is defined as the number of correct matches with respect to the number of correspondences [32]. Our experiment results denotes that the VSG representation for retinal images substantially outperforms other topological graph structures in graph matching. In addition, we also plot recall rate against the percentage of structure (graph node) noise with gaussian curve fit of data in Figure 9. As can be seen, the VSG has obtained relatively high matching rate even with greater than 80% of outliers.

### 4.2. Overall Performance of GM-ICP Algorithm

Evaluating the accuracy of the retinal image registration is not an easy task because of lack of ground truth. In this section, we evaluate our algorithm with three assessment criteria:

- normalized Correlation Coefficient (NCC),
- normalized Mutual Information (NMI),
- vessel Centerline Error Measure (CEM).

The first two terms measure appearance difference between reference and floating retinal images based on intensity statistics. The third term is a standard evaluation criteria that commonly adopted in the field of retinal image analysis [3, 13]. The CEM is defined as median distances over all corresponding points between two vessel centerline models, expressed as

(15) $$\begin{array}{c} \underset{(p_{i},q_{i})\in C}{\text{median}}\left|p_{i}-T\left(q_{i},\theta \right)\right|, \end{array}$$

where *p*_*i*_ and *q*_*i*_ are two matched vessel points determined by correspondence set *C*. *T*(·, *θ*) denotes the final transformation function. In addition, the success rate and run time performance was investigated in our experiments as well. In our settings, a registration was considered successful if the CEM error is smaller than 3.0 pixels. A manual validation was also performed in order to ensure the correctness of our results.

In the second experiment, we compare the performance of GM-ICP with other two registration algorithms: the standard ICP and GDB-ICP. The standard ICP was implemented in Matlab and we try to use it to register two-vessel shape models without initial alignment. The GDB-ICP [8] is a generalized version of DB-ICP which was originally proposed for retinal image registration. We had obtained a public copy of its binary program from the Internet and tested it under the quadratic transformation parameters setting. In order to assess the reliability of the graph matching algorithm, we also performed some image registration tests using the graph matching (GM) alone without ICP.

The results of comparative experiments on 48 retinal image pairs are listed in Table 2. As can be seen, the registration accuracy of GM-ICP (0.81 pixels in CEM) is very close to GDB-ICP (0.79 pixels in CEM) according to three error assessment criteria. Both of the two algorithms have achieved subpixel accuracy. In absence of fine level alignment, the registration with only graph-matching is still robust and obtained acceptable results (1.00 pixels in CEM). The GM-ICP successfully registered 95.83% of all pairs of test retinal images, two pairs failed due to poor quality of vessel segmentation from pathology images whereas GDB-ICP registered 97.92% and only one pair failed. It takes about 8 seconds on average to register a pair of images with GM-ICP, including the time cost in both feature extraction and registration, while GDB-ICP needs more than 20 seconds. In contrast with above three methods, the performance of standard ICP is not satisfactory in both registration accuracy and success rate. Besides, it is a little surprise to see that the NCC and NMI assessment of standard ICP is even better than the other three methods. It denotes that the intensity-based global similarity measures for retinal images are nonconvex and sometimes become unreliable as a result of the presence of large-scale misalignment and inconsistent patterns.

**Figure 10:**
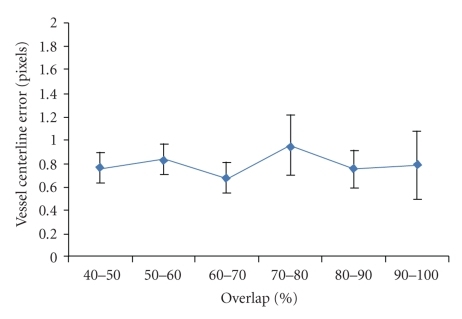
The vessel centerline error measure is plotted against the percentage of image overlap with GM-ICP.

In most of the cases, the performance of registration will be gradually degraded with less overlap of pair of images. We plot the vessel centerline error measure against the percentage of image overlap, as shown in Figure 10. It is observed that GM-ICP is accurate and robust with medium overlapped image pairs. Due to the fact that the graph matching scheme does not rely on direct distance metric between two feature sets, the GM-ICP registration framework is actually invariant to any linear geometric transformations, such as large-scale initial translations and rotations. However, our method may fail with some low overlapped (under 40%) image pairs due to lack of enough consistent vascular segments for matching.

**Figure 11:**
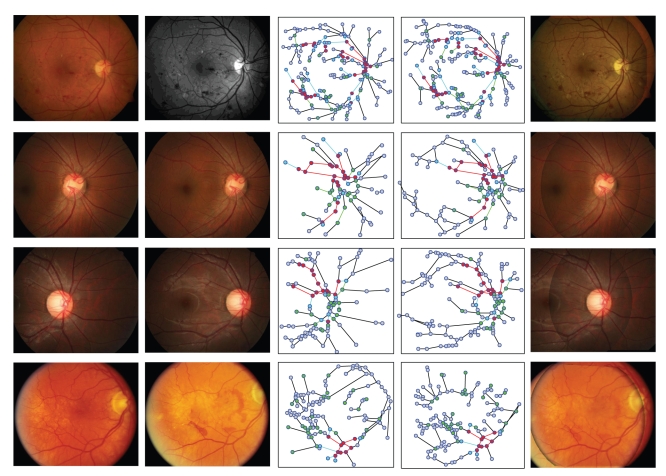
Some image registration results with GM-ICP, from left to right each column represents reference image, floating image, reference VSG, floating VSG, and fused image, respectively.

One difficulty to all vessel-based registration methods is that the reliability of registration is highly influenced by the vessel segmentation results. In our method, there are serval ways to deal with segmentation noise and structure inconsistency. First, small edges were removed during vascular graph representation according to some vessel network topological modification rules [15]. Second, a structure-based sample consensus algorithm was proposed to eliminate incorrect matches in global registration. Third, a point pair weighting strategy is used to reject inconsistent matches during fine level registration. The performance of GM-ICP for the images with high structure noise and pathologies is visually demonstrated in Figure 11.

## 5. Conclusion

In this paper, we proposed a graph-based algorithm framework for retinal image registration. Hierarchical retinal features were utilized in the framework, including vessel shape models, vascular bifurcations and the underlying vascular topological structures. The core idea behind our method is to obtain the point correspondences by taking into account structure information provided by human retina and retinal vessels. Thus, the image registration problem is transformed into a pairwise (edge-to-edge) correspondence problem. Accordingly, we have employed a robust graph matching framework, the graduated assignment with normalization on compatibility matrix, to match two retinal vascular structure graphs. In order to make our method robust to structure noise, the STRUCT-SAC algorithm is proposed to eliminate incorrect matches from graph matching results. The effectiveness of our method has been demonstrated experimentally with 48 pairs of real retinal fundus images.

The VSG representation for retinal vessel network is distinct, preferable spread and low computational cost. Additionally, it reflects the anatomical characterizations contained within a retinal image, which is prominent and almost unchangeable. Our experiments shows that the VSG model is robust even with high structure noise and substantially outperforms other regular topological structures for graph matching.

The most significant distinguishing property of our method is the structure matching and verification scheme for retinal image registration. Although graph matching have proven to be useful in many applications, such as character recognition, shape analysis [29], however, it is relatively rare for medical image registration. The reasons are twofold: (1) structure representation from image is not straightforward; (2) structure matching is not robust under high noise level. In our method, sufficient consistent structures are extracted from retinal images with proper selection of vessel segmentation and graph representation methods. The adopted graph matching framework incorporating with STRUCT-SAC works fine with retinal vascular structure graphs. No initial guess is needed. Heavy local feature descriptors are not required (actually, only two simple distance measures are involved in the computation). Furthermore, it gives a convenient and practical way to distinguish between correct and incorrect structure correspondences.

The main limitation of the proposed method is the assumption that there are enough consistent vascular segments (edges) for matching. However, with some low overlapped or poor quality pathology image pairs, this assumption is not satisfied. As a result, the performance becomes poor (the number of stable graph nodes is less than 3) and even fails.

In this paper, we only addressed pairwise structure matching problem with retinal image registration. It would be interesting to cope with some higher-order graph matching methods [33] in our framework. As a further development, the registration accuracy can be improved by using more efficient and reliable vessel segmentation techniques. Also, our future work will focus on developing more robust graph representation for retinal features, which can accommodate both geometric and photometric invariants in an image.

## Figures and Tables

**Table 1:** The comparative experiment results of five topological graphs for graph matching.

	VSG		DT		DT-MST		KNN		KNN-MST	
Criteria	Mean	Std.	Mean	Std.	Mean	Std.	Mean	Std.	Mean	Std.
Avg Matches Num	18.68	11.57	11.28	9.74	9.46	10.21	8.46	8.43	9.00	9.91
Recall (%)	44.54	17.69	18.19	13.23	14.90	12.85	14.05	11.86	14.03	12.38

Success Rate (%)	95.83		75.00		70.83		70.83		72.92	

**Table 2:** The experiment results of GM-ICP in comparison with GDB-ICP and standard ICP.

	GM		GM-ICP		GDB-ICP		ICP	
Criteria	Mean	Std.	Mean	Std.	Mean	Std.	Mean	Std.
CEM (pixels)	1.0006	0.4953	0.8052	0.2826	0.7890	0.3229	2.6633	2.5355
NMI	0.8985	0.1316	0.9151	0.1103	0.9171	0.1092	0.9529	0.0551
NCC	−0.8978	0.1592	−0.9433	0.0603	−0.9445	0.0601	−0.9697	0.0184

Success Rate (%)	95.83		95.83		97.92		45.83	

Avg Run Time (s)	6.64		7.75		21.57		10.99	
